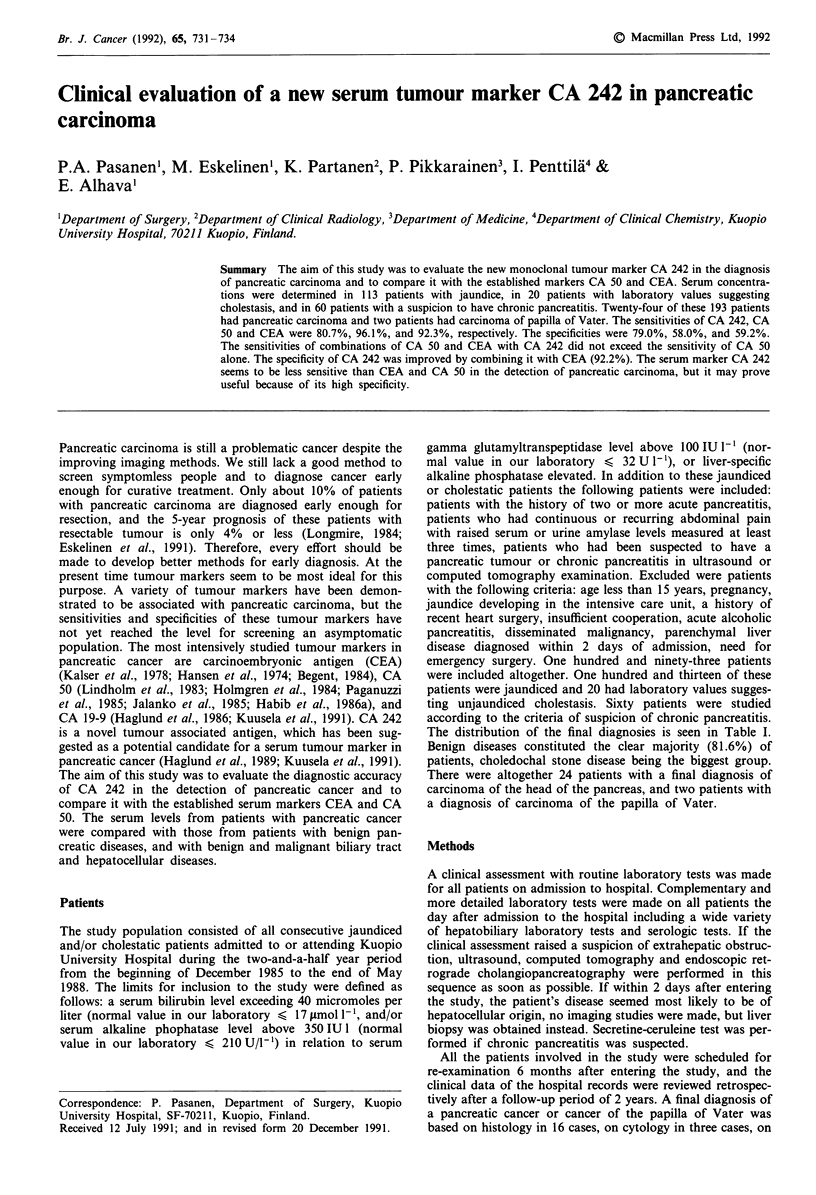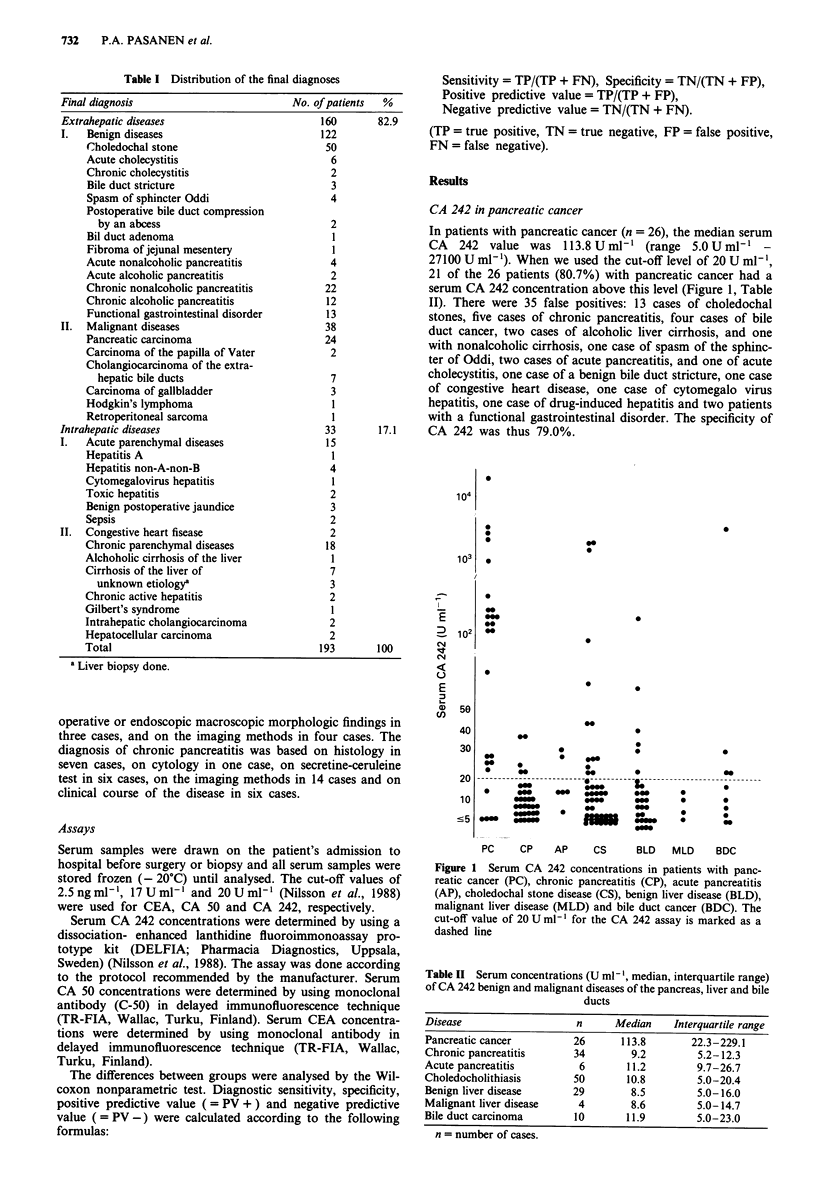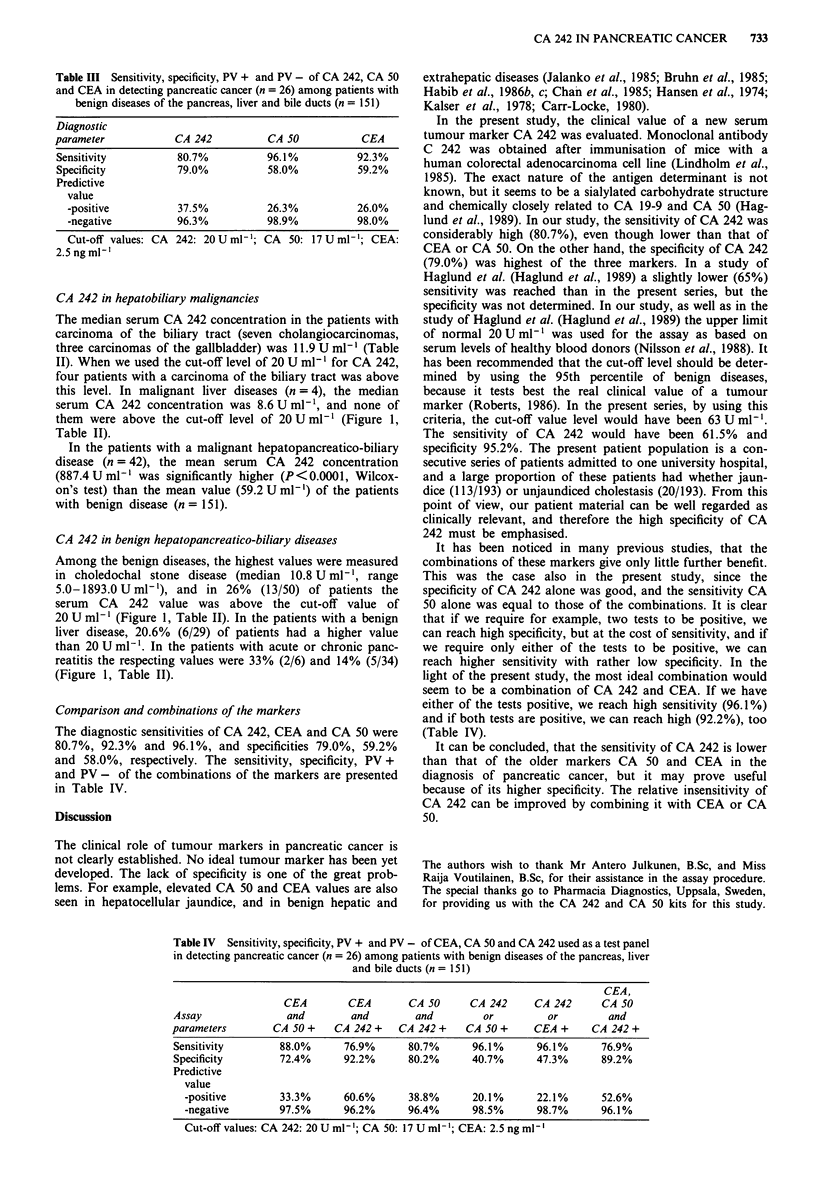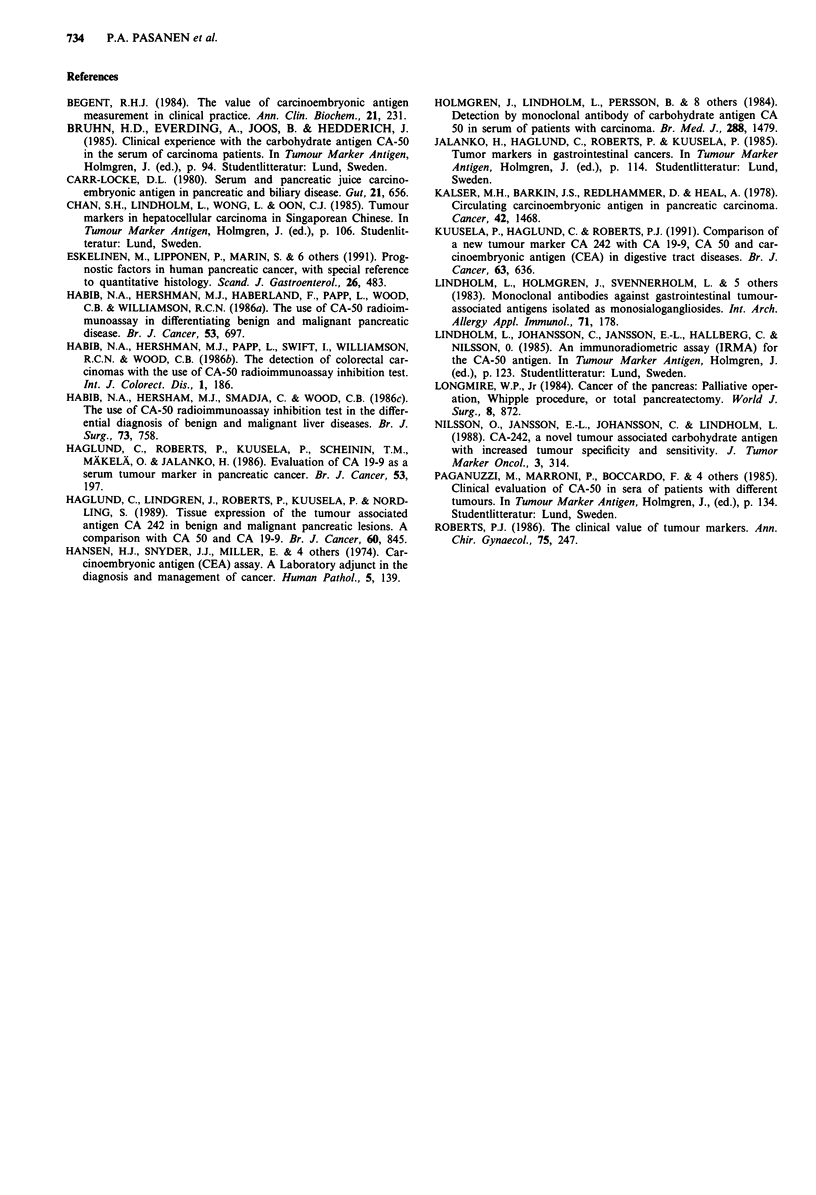# Clinical evaluation of a new serum tumour marker CA 242 in pancreatic carcinoma.

**DOI:** 10.1038/bjc.1992.154

**Published:** 1992-05

**Authors:** P. A. Pasanen, M. Eskelinen, K. Partanen, P. Pikkarainen, I. Penttilä, E. Alhava

**Affiliations:** Department of Surgery, Kuopio University Hospital, Finland.

## Abstract

The aim of this study was to evaluate the new monoclonal tumour marker CA 242 in the diagnosis of pancreatic carcinoma and to compare it with the established markers CA 50 and CEA. Serum concentrations were determined in 113 patients with jaundice, in 20 patients with laboratory values suggesting cholestasis, and in 60 patients with a suspicion to have chronic pancreatitis. Twenty-four of these 193 patients had pancreatic carcinoma and two patients had carcinoma of papilla of Vater. The sensitivities of CA 242, CA 50 and CEA were 80.7%, 96.1%, and 92.3%, respectively. The specificities were 79.0%, 58.0%, and 59.2%. The sensitivities of combinations of CA 50 and CEA with CA 242 did not exceed the sensitivity of CA 50 alone. The specificity of CA 242 was improved by combining it with CEA (92.2%). The serum marker CA 242 seems to be less sensitive than CEA and CA 50 in the detection of pancreatic carcinoma, but it may prove useful because of its high specificity.


Br. J. Cancer (1992), 65, 731-734 © Macmillan Press Ltd, 1992

### Clinical evaluation of a new serum tumour marker CA 242 in pancreatic carcinoma

P.A. Pasanen[1], M. Eskelinen[1], K. Partanen[2], P. Pikkarainen[3], I. Penttilä[4] & E. Alhava[1]

[1]*Department of Surgery,* [2]*Department of Clinical Radiology,* [3]*Department of Medicine,* [4]*Department of Clinical Chemistry, Kuopio University Hospital, 70211 Kuopio, Finland.*

**Summary** The aim of this study was to evaluate the new monoclonal tumour marker CA 242 in the diagnosis of pancreatic carcinoma and to compare it with the established markers CA 50 and CEA. Serum concentrations were determined in 113 patients with jaundice, in 20 patients with laboratory values suggesting cholestasis, and in 60 patients with a suspicion to have chronic pancreatitis. Twenty-four of these 193 patients had pancreatic carcinoma and two patients had carcinoma of papilla of Vater. The sensitivities of CA 242, CA 50 and CEA were 80.7%, 96.1%, and 92.3%, respectively. The specificities were 79.0%, 58.0%, and 59.2%. The sensitivities of combinations of CA 50 and CEA with CA 242 did not exceed the sensitivity of CA 50 alone. The specificity of CA 242 was improved by combining it with CEA (92.2%). The serum marker CA 242 seems to be less sensitive than CEA and CA 50 in the detection of pancreatic carcinoma, but it may prove useful because of its high specificity.

Pancreatic carcinoma is still a problematic cancer despite the improving imaging methods. We still lack a good method to screen symptomless people and to diagnose cancer early enough for curative treatment. Only about 10% of patients with pancreatic carcinoma are diagnosed early enough for resection, and the 5-year prognosis of these patients with resectable tumour is only 4% or less (Longmire, 1984; Eskelinen *et al.*, 1991). Therefore, every effort should be made to develop better methods for early diagnosis. At the present time tumour markers seem to be most ideal for this purpose. A variety of tumour markers have been demonstrated to be associated with pancreatic carcinoma, but the sensitivities and specificities of these tumour markers have not yet reached the level for screening an asymptomatic population. The most intensively studied tumour markers in pancreatic cancer are carcinoembryonic antigen (CEA) (Kalser *et al.*, 1978; Hansen *et al.*, 1974; Begent, 1984), CA 50 (Lindholm *et al.*, 1983; Holmgren *et al.*, 1984; Paganuzzi *et al.*, 1985; Jalanko *et al.*, 1985; Habib *et al.*, 1986a), and CA 19-9 (Haglund *et al.*, 1986; Kuusela *et al.*, 1991). CA 242 is a novel tumour associated antigen, which has been suggested as a potential candidate for a serum tumour marker in pancreatic cancer (Haglund *et al.*, 1989; Kuusela *et al.*, 1991). The aim of this study was to evaluate the diagnostic accuracy of CA 242 in the detection of pancreatic cancer and to compare it with the established serum markers CEA and CA 50. The serum levels from patients with pancreatic cancer were compared with those from patients with benign pancreatic diseases, and with benign and malignant biliary tract and hepatocellular diseases.

### Patients

The study population consisted of all consecutive jaundiced and/or cholestatic patients admitted to or attending Kuopio University Hospital during the two-and-a-half year period from the beginning of December 1985 to the end of May 1988. The limits for inclusion to the study were defined as follows: a serum bilirubin level exceeding 40 micromoles per liter (normal value in our laboratory $\leqslant 17\ \mu mol\ l^{-1}$, and/or serum alkaline phophatase level above 350 IU l (normal value in our laboratory $\leqslant 210\ U/l^{-1}$) in relation to serum gamma glutamyltranspeptidase level above $100\ IU\ l^{-1}$ (normal value in our laboratory $\leqslant 32\ U\ l^{-1}$), or liver-specific alkaline phosphatase elevated. In addition to these jaundiced or cholestatic patients the following patients were included: patients with the history of two or more acute pancreatitis, patients who had continuous or recurring abdominal pain with raised serum or urine amylase levels measured at least three times, patients who had been suspected to have a pancreatic tumour or chronic pancreatitis in ultrasound or computed tomography examination. Excluded were patients with the following criteria: age less than 15 years, pregnancy, jaundice developing in the intensive care unit, a history of recent heart surgery, insufficient cooperation, acute alcoholic pancreatitis, disseminated malignancy, parenchymal liver disease diagnosed within 2 days of admission, need for emergency surgery. One hundred and ninety-three patients were included altogether. One hundred and thirteen of these patients were jaundiced and 20 had laboratory values suggesting unjaundiced cholestasis. Sixty patients were studied according to the criteria of suspicion of chronic pancreatitis. The distribution of the final diagnosies is seen in Table I. Benign diseases constituted the clear majority (81.6%) of patients, choledochal stone disease being the biggest group. There were altogether 24 patients with a final diagnosis of carcinoma of the head of the pancreas, and two patients with a diagnosis of carcinoma of the papilla of Vater.

### Methods

A clinical assessment with routine laboratory tests was made for all patients on admission to hospital. Complementary and more detailed laboratory tests were made on all patients the day after admission to the hospital including a wide variety of hepatobiliary laboratory tests and serologic tests. If the clinical assessment raised a suspicion of extrahepatic obstruction, ultrasound, computed tomography and endoscopic retrograde cholangiopancreatography were performed in this sequence as soon as possible. If within 2 days after entering the study, the patient's disease seemed most likely to be of hepatocellular origin, no imaging studies were made, but liver biopsy was obtained instead. Secretine-ceruleine test was performed if chronic pancreatitis was suspected.

All the patients involved in the study were scheduled for re-examination 6 months after entering the study, and the clinical data of the hospital records were reviewed retrospectively after a follow-up period of 2 years. A final diagnosis of a pancreatic cancer or cancer of the papilla of Vater was based on histology in 16 cases, on cytology in three cases, on

Correspondence: P. Pasanen, Department of Surgery, Kuopio University Hospital, SF-70211, Kuopio, Finland.
Received 12 July 1991; and in revised form 20 December 1991.

**Table I** Distribution of the final diagnoses

| *Final diagnosis* | *No. of patients* | *%* |
|---|---|---|
| *Extrahepatic diseases* | 160 | 82.9 |
| I. Benign diseases | 122 | |
| Choledochal stone | 50 | |
| Acute cholecystitis | 6 | |
| Chronic cholecystitis | 2 | |
| Bile duct stricture | 3 | |
| Spasm of sphincter Oddi | 4 | |
| Postoperative bile duct compression by an abcess | 2 | |
| Bil duct adenoma | 1 | |
| Fibroma of jejunal mesentery | 1 | |
| Acute nonalcoholic pancreatitis | 4 | |
| Acute alcoholic pancreatitis | 2 | |
| Chronic nonalcoholic pancreatitis | 22 | |
| Chronic alcoholic pancreatitis | 12 | |
| Functional gastrointestinal disorder | 13 | |
| II. Malignant diseases | 38 | |
| Pancreatic carcinoma | 24 | |
| Carcinoma of the papilla of Vater | 2 | |
| Cholangiocarcinoma of the extra-hepatic bile ducts | 7 | |
| Carcinoma of gallbladder | 3 | |
| Hodgkin's lymphoma | 1 | |
| Retroperitoneal sarcoma | 1 | |
| *Intrahepatic diseases* | 33 | 17.1 |
| I. Acute parenchymal diseases | 15 | |
| Hepatitis A | 1 | |
| Hepatitis non-A-non-B | 4 | |
| Cytomegalovirus hepatitis | 1 | |
| Toxic hepatitis | 2 | |
| Benign postoperative jaundice | 3 | |
| Sepsis | 2 | |
| II. Congestive heart fisease | 2 | |
| Chronic parenchymal diseases | 18 | |
| Alchoholic cirrhosis of the liver | 1 | |
| Cirrhosis of the liver of | 7 | |
| unknown etiology[a] | 3 | |
| Chronic active hepatitis | 2 | |
| Gilbert's syndrome | 1 | |
| Intrahepatic cholangiocarcinoma | 2 | |
| Hepatocellular carcinoma | 2 | |
| Total | 193 | 100 |

[a] Liver biopsy done.

operative or endoscopic macroscopic morphologic findings in three cases, and on the imaging methods in four cases. The diagnosis of chronic pancreatitis was based on histology in seven cases, on cytology in one case, on secretine-ceruleine test in six cases, on the imaging methods in 14 cases and on clinical course of the disease in six cases.

*Assays*

Serum samples were drawn on the patient's admission to hospital before surgery or biopsy and all serum samples were stored frozen (− 20°C) until analysed. The cut-off values of 2.5 ng $ml^{-1}$, 17 U $ml^{-1}$ and 20 U $ml^{-1}$ (Nilsson *et al.*, 1988) were used for CEA, CA 50 and CA 242, respectively.

Serum CA 242 concentrations were determined by using a dissociation- enhanced lanthidine fluoroimmonoassay prototype kit (DELFIA; Pharmacia Diagnostics, Uppsala, Sweden) (Nilsson *et al.*, 1988). The assay was done according to the protocol recommended by the manufacturer. Serum CA 50 concentrations were determined by using monoclonal antibody (C-50) in delayed immunofluorescence technique (TR-FIA, Wallac, Turku, Finland). Serum CEA concentrations were determined by using monoclonal antibody in delayed immunofluorescence technique (TR-FIA, Wallac, Turku, Finland).

The differences between groups were analysed by the Wilcoxon nonparametric test. Diagnostic sensitivity, specificity, positive predictive value ( = PV + ) and negative predictive value ( = PV − ) were calculated according to the following formulas:

Sensitivity = TP/(TP + FN), Specificity = TN/(TN + FP),
Positive predictive value = TP/(TP + FP),
Negative predictive value = TN/(TN + FN).

(TP = true positive, TN = true negative, FP = false positive, FN = false negative).

### Results

*CA 242 in pancreatic cancer*

In patients with pancreatic cancer ($n$ = 26), the median serum CA 242 value was 113.8 U $ml^{-1}$ (range 5.0 U $ml^{-1}$ – 27100 U $ml^{-1}$). When we used the cut-off level of 20 U $ml^{-1}$, 21 of the 26 patients (80.7%) with pancreatic cancer had a serum CA 242 concentration above this level (Figure 1, Table II). There were 35 false positives: 13 cases of choledochal stones, five cases of chronic pancreatitis, four cases of bile duct cancer, two cases of alcoholic liver cirrhosis, and one with nonalcoholic cirrhosis, one case of spasm of the sphincter of Oddi, two cases of acute pancreatitis, and one of acute cholecystitis, one case of a benign bile duct stricture, one case of congestive heart disease, one case of cytomegalo virus hepatitis, one case of drug-induced hepatitis and two patients with a functional gastrointestinal disorder. The specificity of CA 242 was thus 79.0%.

**Figure 1** Serum CA 242 concentrations in patients with pancreatic cancer (PC), chronic pancreatitis (CP), acute pancreatitis (AP), choledochal stone disease (CS), benign liver disease (BLD), malignant liver disease (MLD) and bile duct cancer (BDC). The cut-off value of 20 U $ml^{-1}$ for the CA 242 assay is marked as a dashed line

**Table II** Serum concentrations (U $ml^{-1}$, median, interquartile range) of CA 242 benign and malignant diseases of the pancreas, liver and bile ducts

| *Disease* | *n* | *Median* | *Interquartile range* |
|---|---|---|---|
| Pancreatic cancer | 26 | 113.8 | 22.3–229.1 |
| Chronic pancreatitis | 34 | 9.2 | 5.2–12.3 |
| Acute pancreatitis | 6 | 11.2 | 9.7–26.7 |
| Choledocholithiasis | 50 | 10.8 | 5.0–20.4 |
| Benign liver disease | 29 | 8.5 | 5.0–16.0 |
| Malignant liver disease | 4 | 8.6 | 5.0–14.7 |
| Bile duct carcinoma | 10 | 11.9 | 5.0–23.0 |

$n$ = number of cases.

**Table III** Sensitivity, specificity, PV + and PV – of CA 242, CA 50 and CEA in detecting pancreatic cancer ($n = 26$) among patients with benign diseases of the pancreas, liver and bile ducts ($n = 151$)

| *Diagnostic parameter* | *CA 242* | *CA 50* | *CEA* |
|---|---|---|---|
| Sensitivity | 80.7% | 96.1% | 92.3% |
| Specificity | 79.0% | 58.0% | 59.2% |
| Predictive value | | | |
| -positive | 37.5% | 26.3% | 26.0% |
| -negative | 96.3% | 98.9% | 98.0% |

Cut-off values: CA 242: 20 U ml$^{-1}$; CA 50: 17 U ml$^{-1}$; CEA: 2.5 ng ml$^{-1}$

*CA 242 in hepatobiliary malignancies*

The median serum CA 242 concentration in the patients with carcinoma of the biliary tract (seven cholangiocarcinomas, three carcinomas of the gallbladder) was 11.9 U ml$^{-1}$ (Table II). When we used the cut-off level of 20 U ml$^{-1}$ for CA 242, four patients with a carcinoma of the biliary tract was above this level. In malignant liver diseases ($n = 4$), the median serum CA 242 concentration was 8.6 U ml$^{-1}$, and none of them were above the cut-off level of 20 U ml$^{-1}$ (Figure 1, Table II).

In the patients with a malignant hepatopancreatico-biliary disease ($n = 42$), the mean serum CA 242 concentration (887.4 U ml$^{-1}$ was significantly higher ($P < 0.0001$, Wilcoxon's test) than the mean value (59.2 U ml$^{-1}$) of the patients with benign disease ($n = 151$).

*CA 242 in benign hepatopancreatico-biliary diseases*

Among the benign diseases, the highest values were measured in choledochal stone disease (median 10.8 U ml$^{-1}$, range 5.0–1893.0 U ml$^{-1}$), and in 26% (13/50) of patients the serum CA 242 value was above the cut-off value of 20 U ml$^{-1}$ (Figure 1, Table II). In the patients with a benign liver disease, 20.6% (6/29) of patients had a higher value than 20 U ml$^{-1}$. In the patients with acute or chronic pancreatitis the respecting values were 33% (2/6) and 14% (5/34) (Figure 1, Table II).

*Comparison and combinations of the markers*

The diagnostic sensitivities of CA 242, CEA and CA 50 were 80.7%, 92.3% and 96.1%, and specificities 79.0%, 59.2% and 58.0%, respectively. The sensitivity, specificity, PV + and PV – of the combinations of the markers are presented in Table IV.

### Discussion

The clinical role of tumour markers in pancreatic cancer is not clearly established. No ideal tumour marker has been yet developed. The lack of specificity is one of the great problems. For example, elevated CA 50 and CEA values are also seen in hepatocellular jaundice, and in benign hepatic and extrahepatic diseases (Jalanko *et al.*, 1985; Bruhn *et al.*, 1985; Habib *et al.*, 1986*b*, *c*; Chan *et al.*, 1985; Hansen *et al.*, 1974; Kalser *et al.*, 1978; Carr-Locke, 1980).

In the present study, the clinical value of a new serum tumour marker CA 242 was evaluated. Monoclonal antibody C 242 was obtained after immunisation of mice with a human colorectal adenocarcinoma cell line (Lindholm *et al.*, 1985). The exact nature of the antigen determinant is not known, but it seems to be a sialylated carbohydrate structure and chemically closely related to CA 19-9 and CA 50 (Haglund *et al.*, 1989). In our study, the sensitivity of CA 242 was considerably high (80.7%), even though lower than that of CEA or CA 50. On the other hand, the specificity of CA 242 (79.0%) was highest of the three markers. In a study of Haglund *et al.* (Haglund *et al.*, 1989) a slightly lower (65%) sensitivity was reached than in the present series, but the specificity was not determined. In our study, as well as in the study of Haglund *et al.* (Haglund *et al.*, 1989) the upper limit of normal 20 U ml$^{-1}$ was used for the assay as based on serum levels of healthy blood donors (Nilsson *et al.*, 1988). It has been recommended that the cut-off level should be determined by using the 95th percentile of benign diseases, because it tests best the real clinical value of a tumour marker (Roberts, 1986). In the present series, by using this criteria, the cut-off value level would have been 63 U ml$^{-1}$. The sensitivity of CA 242 would have been 61.5% and specificity 95.2%. The present patient population is a consecutive series of patients admitted to one university hospital, and a large proportion of these patients had whether jaundice (113/193) or unjaundiced cholestasis (20/193). From this point of view, our patient material can be well regarded as clinically relevant, and therefore the high specificity of CA 242 must be emphasised.

It has been noticed in many previous studies, that the combinations of these markers give only little further benefit. This was the case also in the present study, since the specificity of CA 242 alone was good, and the sensitivity CA 50 alone was equal to those of the combinations. It is clear that if we require for example, two tests to be positive, we can reach high specificity, but at the cost of sensitivity, and if we require only either of the tests to be positive, we can reach higher sensitivity with rather low specificity. In the light of the present study, the most ideal combination would seem to be a combination of CA 242 and CEA. If we have either of the tests positive, we reach high sensitivity (96.1%) and if both tests are positive, we can reach high (92.2%), too (Table IV).

It can be concluded, that the sensitivity of CA 242 is lower than that of the older markers CA 50 and CEA in the diagnosis of pancreatic cancer, but it may prove useful because of its higher specificity. The relative insensitivity of CA 242 can be improved by combining it with CEA or CA 50.

The authors wish to thank Mr Antero Julkunen, B.Sc, and Miss Raija Voutilainen, B.Sc, for their assistance in the assay procedure. The special thanks go to Pharmacia Diagnostics, Uppsala, Sweden, for providing us with the CA 242 and CA 50 kits for this study.

**Table IV** Sensitivity, specificity, PV + and PV – of CEA, CA 50 and CA 242 used as a test panel in detecting pancreatic cancer ($n = 26$) among patients with benign diseases of the pancreas, liver and bile ducts ($n = 151$)

| *Assay parameters* | *CEA and CA 50 +* | *CEA and CA 242 +* | *CA 50 and CA 242 +* | *CA 242 or CA 50 +* | *CA 242 or CEA +* | *CEA, CA 50 and CA 242 +* |
|---|---|---|---|---|---|---|
| Sensitivity | 88.0% | 76.9% | 80.7% | 96.1% | 96.1% | 76.9% |
| Specificity | 72.4% | 92.2% | 80.2% | 40.7% | 47.3% | 89.2% |
| Predictive value | | | | | | |
| -positive | 33.3% | 60.6% | 38.8% | 20.1% | 22.1% | 52.6% |
| -negative | 97.5% | 96.2% | 96.4% | 98.5% | 98.7% | 96.1% |

Cut-off values: CA 242: 20 U ml$^{-1}$; CA 50: 17 U ml$^{-1}$; CEA: 2.5 ng ml$^{-1}$

### References

BEGENT, R.H.J. (1984). The value of carcinoembryonic antigen measurement in clinical practice. *Ann. Clin. Biochem.*, **21**, 231.

BRUHN, H.D., EVERDING, A., JOOS, B. & HEDDERICH, J. (1985). Clinical experience with the carbohydrate antigen CA-50 in the serum of carcinoma patients. In *Tumour Marker Antigen*, Holmgren, J. (ed.), p. 94. Studentlitteratur: Lund, Sweden.

CARR-LOCKE, D.L. (1980). Serum and pancreatic juice carcinoembryonic antigen in pancreatic and biliary disease. *Gut*, **21**, 656.

CHAN, S.H., LINDHOLM, L., WONG, L. & OON, C.J. (1985). Tumour markers in hepatocellular carcinoma in Singaporean Chinese. In *Tumour Marker Antigen*, Holmgren, J. (ed.), p. 106. Studenlitteratur: Lund, Sweden.

ESKELINEN, M., LIPPONEN, P., MARIN, S. & 6 others (1991). Prognostic factors in human pancreatic cancer, with special reference to quantitative histology. *Scand. J. Gastroenterol.*, **26**, 483.

HABIB, N.A., HERSHMAN, M.J., HABERLAND, F., PAPP, L., WOOD, C.B. & WILLIAMSON, R.C.N. (1986*a*). The use of CA-50 radioimmunoassay in differentiating benign and malignant pancreatic disease. *Br. J. Cancer*, **53**, 697.

HABIB, N.A., HERSHMAN, M.J., PAPP, L., SWIFT, I., WILLIAMSON, R.C.N. & WOOD, C.B. (1986*b*). The detection of colorectal carcinomas with the use of CA-50 radioimmunoassay inhibition test. *Int. J. Colorect. Dis.*, **1**, 186.

HABIB, N.A., HERSHAM, M.J., SMADJA, C. & WOOD, C.B. (1986*c*). The use of CA-50 radioimmunoassay inhibition test in the differential diagnosis of benign and malignant liver diseases. *Br. J. Surg.*, **73**, 758.

HAGLUND, C., ROBERTS, P., KUUSELA, P., SCHEININ, T.M., MÄKELÄ, O. & JALANKO, H. (1986). Evaluation of CA 19-9 as a serum tumour marker in pancreatic cancer. *Br. J. Cancer*, **53**, 197.

HAGLUND, C., LINDGREN, J., ROBERTS, P., KUUSELA, P. & NORDLING, S. (1989). Tissue expression of the tumour associated antigen CA 242 in benign and malignant pancreatic lesions. A comparison with CA 50 and CA 19-9. *Br. J. Cancer*, **60**, 845.

HANSEN, H.J., SNYDER, J.J., MILLER, E. & 4 others (1974). Carcinoembryonic antigen (CEA) assay. A Laboratory adjunct in the diagnosis and management of cancer. *Human Pathol.*, **5**, 139.

HOLMGREN, J., LINDHOLM, L., PERSSON, B. & 8 others (1984). Detection by monoclonal antibody of carbohydrate antigen CA 50 in serum of patients with carcinoma. *Br. Med. J.*, **288**, 1479.

JALANKO, H., HAGLUND, C., ROBERTS, P. & KUUSELA, P. (1985). Tumor markers in gastrointestinal cancers. In *Tumour Marker Antigen*, Holmgren, J. (ed.), p. 114. Studentlitteratur: Lund, Sweden.

KALSER, M.H., BARKIN, J.S., REDLHAMMER, D. & HEAL, A. (1978). Circulating carcinoembryonic antigen in pancreatic carcinoma. *Cancer*, **42**, 1468.

KUUSELA, P., HAGLUND, C. & ROBERTS, P.J. (1991). Comparison of a new tumour marker CA 242 with CA 19-9, CA 50 and carcinoembryonic antigen (CEA) in digestive tract diseases. *Br. J. Cancer*, **63**, 636.

LINDHOLM, L., HOLMGREN, J., SVENNERHOLM, L. & 5 others (1983). Monoclonal antibodies against gastrointestinal tumour-associated antigens isolated as monosialogangliosides. *Int. Arch. Allergy Appl. Immunol.*, **71**, 178.

LINDHOLM, L., JOHANSSON, C., JANSSON, E.-L., HALLBERG, C. & NILSSON, 0. (1985). An immunoradiometric assay (IRMA) for the CA-50 antigen. In *Tumour Marker Antigen*, Holmgren, J. (ed.), p. 123. Studentlitteratur: Lund, Sweden.

LONGMIRE, W.P., Jr (1984). Cancer of the pancreas: Palliative operation, Whipple procedure, or total pancreatectomy. *World J. Surg.*, **8**, 872.

NILSSON, O., JANSSON, E.-L., JOHANSSON, C. & LINDHOLM, L. (1988). CA-242, a novel tumour associated carbohydrate antigen with increased tumour specificity and sensitivity. *J. Tumor Marker Oncol.*, **3**, 314.

PAGANUZZI, M., MARRONI, P., BOCCARDO, F. & 4 others (1985). Clinical evaluation of CA-50 in sera of patients with different tumours. In *Tumour Marker Antigen*, Holmgren, J., (ed.), p. 134. Studentlitteratur: Lund, Sweden.

ROBERTS, P.J. (1986). The clinical value of tumour markers. *Ann. Chir. Gynaecol.*, **75**, 247.